# Anti-Proliferative Effects of Standardized *Cornus officinalis* on Benign Prostatic Epithelial Cells via the PCNA/E2F1-Dependent Cell Cycle Pathway

**DOI:** 10.3390/ijms21249567

**Published:** 2020-12-15

**Authors:** Bo-Ram Jin, Se-Yun Cheon, Hyo-Jung Kim, Myoung-Seok Kim, Kwang-Ho Lee, Hyo-Jin An

**Affiliations:** 1 Department of Pharmacology, College of Korean Medicine, Sang-ji University, Wonju-si, Gangwon-do 26339, Korea; wlsqh92@gmail.com (B.-R.J.); chunsay1008@naver.com (S.-Y.C.); hyojung_95@naver.com (H.-J.K.); 2 AmcoBio Inc., 1789 Nambusunhwan-ro, Gwanak-gu, Seoul 08758, Korea; 3 Central Research of Hawon Pharmaceutical, JangHeung 59338, Korea; mskim1210@naver.com (M.-S.K.); gusins0930@naver.com (K.-H.L.)

**Keywords:** benign prostate hyperplasia, cell cycle, *Cornus officinalis* Sieb. et Zucc., E2F1, proliferating cell nuclear antigen, proliferation

## Abstract

*Cornus officinalis*, widely used in traditional Chinese medicine, exhibits pharmacological effects against erectile dysfunction and pollakisuria, which are pathological symptoms of benign prostatic hyperplasia (BPH). Although traditional usage and a study on BPH have been reported, to our knowledge, no study has investigated the exact molecular mechanism(s) underlying the anti-proliferative effects of standardized *C. officinalis* on prostatic cells. We standardized *C. officinalis* 30% ethanol extract (COFE) and demonstrated the therapeutic effects of COFE on human BPH epithelial cells and testosterone-induced BPH in rats. In vitro studies using BPH-1 cells demonstrated an upregulation of BPH-related and E2F Transcription Factor 1(E2F1)-dependent cell cycle markers, whereas treatment with COFE clearly inhibited the proliferation of BPH epithelial cells and reduced the overexpression of G1 and S checkpoint genes. Additionally, COFE administration alleviated the androgen-dependent prostatic enlargement in a testosterone-induced BPH animal model. COFE exerted these anti-BPH effects by the inhibition of anti-apoptotic markers, suppression of PCNA expression, and regulation of E2F1/pRB-dependent cell cycle markers in rats with BPH. These results suggest that COFE exerts anti-proliferative effect by regulating PCNA/E2F1-dependent cell cycle signaling pathway both in vivo and in vitro. These findings reveal the therapeutic potential of COFE, which could be used as a substitute for BPH treatment.

## 1. Introduction

BPH is defined as the enlargement of the prostate gland and is a common disease in aging men. The estimated incidence of BPH is 50% in men aged 50–60 years and 90% in those over 80 years [1]. Histologically, BPH is characterized by a non-malignant proliferation of epithelial and stromal cells in the prostatic glandular tissues [2]. Although the etiology of BPH is not fully elucidated, evidence suggest that male sex hormones and aging are important factors for the development of BPH [3]. Androgenic hormones, testosterone, and dihydrotestosterone (DHT), are important determinants of the pathogenesis of BPH. Therefore, to improve BPH-associated lower urinary tract symptoms, androgen ablation therapy, such as the use of finasteride and other 5α-reductase inhibitors, forms the basis of medication [4]. However, these treatments are not completely successful owing to the risk of adverse reactions, such as fatigue, weakness, insomnia, sexual dysfunction, and prostate cancer. In addition, a clear understanding of its molecular mechanisms is still lacking, suggesting that substitutional agents that could exert more effective functions and reduce side effects in clinical use are required.

Proliferating cell nuclear antigen (PCNA) is the eukaryotic DNA sliding clamp that interacts with cell cycle proteins, including cyclins and Cyclin-dependent kinases (CDKs), involved in DNA replication [5]. Several cyclin-CDK complexes, where cyclin and CDK bind to each other and regulate target proteins, characteristically promote the distinct cell cycle phases [6]. In response to extracellular signals, active cyclin D-CDK4 complex phosphorylates retinoblastoma susceptibility protein (RB) signaling pathway. Subsequently, E2F dissociates from the E2F/DP1/RB complex and then transactivates various S-phase genes, including cyclins A, D, and E. Consistent with the notions that a major role of G1/S cyclin-CDK complexes is to activate E2F, the overexpression of E2F is associated with the induction of S phase [7]. In this regard, studies have found that the E2F1/pRB signaling pathway is also an underlying signaling pathway of prostatic diseases, including BPH [8].

*Cornus officinalis* Sieb. et Zucc., a part of the genus *Cornus* of the family Cornaceae, has been widely used in traditional Chinese medicine (TCM) for over 2000 years. *C. officinalis* is categorized as a yang-tonifying medicine, which nourishes and replenishes the lack of yang qi. In general, “yang-tonifying” is associated with the improvement of sexual performance and male reproductive system.

In TCM, *C. officinalis* has been characterized as an excellent tonic and astringent. In view of that, medicinally *C. officinalis* is used for the treatment of spermatorrhoea and urination disorder. There is a long list of popular herbal formulas that are based on *C. officinalis* [9,10,11]. Recent pharmacological studies have reported that *C. officinalis* and its bioactive constituents exhibit positive effects on the reproductive abilities, such as improving erectile dysfunction and enhancing the motility of human sperm [12]. These biological activities are consistent with those reported for the *C. officinalis* used as traditional medicine, which are TCM formulae to treat urinary frequency and seminal emission [13]. Furthermore, recent study has shown that aqueous extract of *C. officinalis* attenuates testosterone-induced benign prostatic hyperplasia (BPH) by suppressing androgen signaling pathway [14]. However, no study has investigated the exact molecular mechanism(s) underlying the anti-proliferative effects of standardized *C. officinalis* on prostatic cells.

In this study, we explored the effects of COFE on the development and progression of BPH and the involvement in PCNA/E2F1-dependent cell cycle pathway. Using BPH-1 cells, we studied the effect of standardized *C. officinalis* 30% ethanol extract (COFE) on the proliferation of epithelial cells and elucidated the underlying molecular mechanism. In addition, we established the testosterone-induced BPH animal model to investigate the cell cycle regulatory action and anti-proliferative effect by COFE in the prostate tissues.

## 2. Results

### 2.1. Chemical Profiling Analysis of COFE

**Figure 1:**
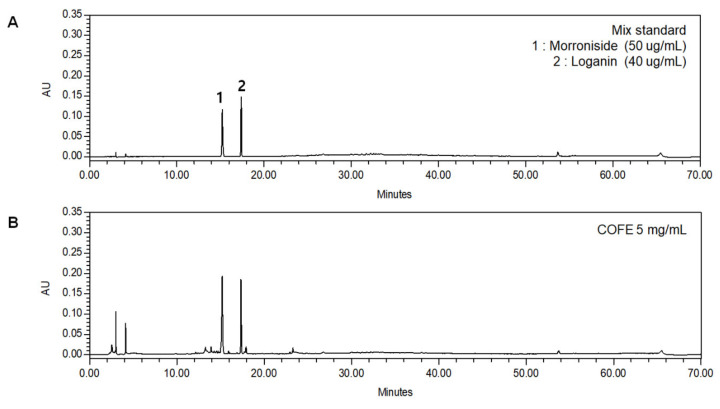
Representative HPLC chromatograms of (**A**) mixed standards, (**B**) *Cornus officinalis*. (1) morroniside; (2) loganin. The two analytes have very broad range of polarity and various mobile phase compositions were tested. The mixture of 10 mM ammonium acetate (*w*/*v*), and acetonitrile was finally chosen as the preferred mobile phase. Based on the ultraviolet absorption characteristics of major compounds, the chromatograms were recorded at a wavelength of 240 nm. The separation was optimized when the column temperature was kept at 35 °C.

Representative spectrometry data of the mixed standards and COFE are shown in Figure 1. The two standard compounds were prepared to establish the calibration curves constructed by plotting the mean peak area versus the standard concentrations. All standard curves show a suitable linear regression (morroniside: *r* = 0.9999; loganin: *r* = 0.9995) over the tested range. The limits of detection (LOD) and limits of quantification (LOQ) were analyzed for each compound. The compounds were identified by comparing their retention time and UV spectra with injected standards under the same conditions. The amount of morroniside in COFE is 7.490 ± 0.143 mg/g (retention time: 15.197 min) and that of loganin is 4.821 ± 0.143 (retention time: 17.368 min).

### 2.2. COFE Inhibited Markers of Cell Proliferation in BPH-1 Cells

**Figure 2:**
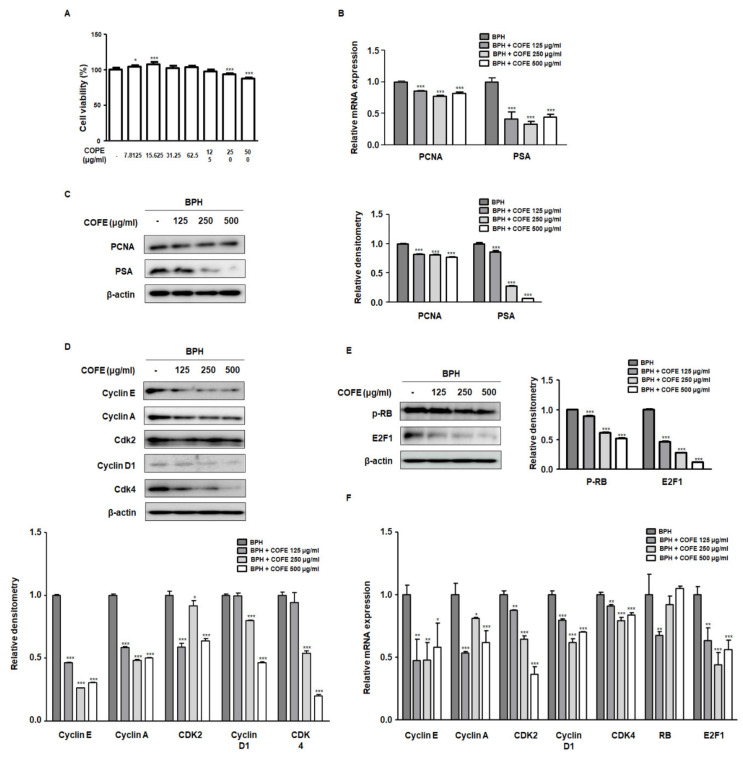
The inhibitory effects of *C. officinalis* 30% ethanol extract (COFE) on benign prostatic hyperplasia (BPH)-1 human BPH epithelial cells. (**A**) BPH-1 cells were treated with various concentrations of COFE for 24 h (7.8125–500 μg/mL) to assess cell viability. (**B**–**F**) The BPH-1 cells were treated with or without COFE 125, 250, and 500 μg/mL. (**B**) The mRNA level of proliferating cell nuclear antigen (PCNA) and prostate specific antigen (PSA) was analyzed by RT-qPCR analysis. BPH-1 cell lysates were immunoblotted with (**C**) PCNA. PSA (**D**) Cyclin E, Cyclin A, Cdk2, Cyclin D1, and Cdk4 (**E**) p-RB, E2F1 primary antibodies. β-actin served as an internal control. Densitometric analysis of each protein was performed and relative protein levels were represented as mean ± SD. (**F**) The mRNA level of Cyclin E, Cyclin A, Cdk2, Cyclin D1, Cdk4, RB, and E2F1 was quantified using RT-qPCR in BPH-1 cells. The Ct values of genes were normalized using the Ct value of GAPDH. * *p* < 0.05, ** *p* < 0.01, *** *p* < 0.001 versus vehicle group.

To evaluate the inhibitory effects of COFE on proliferation of BPH-1 cells, we performed an MTT assay. As shown in Figure 2A, treatment with COFE (125–500 μg/mL) for 24 h abrogated the proliferation of BPH-1 cells. Considering that PCNA and prostate specific antigen (PSA) are important factors of the progression of experimental BPH, we investigated whether COFE inhibited the molecular targets of BPH. PCNA and PSA were overexpressed in BPH-1 cells, whereas treatment of COFE (125, 250, and 500 μg/mL) for 24 h significantly suppressed the expression of PCNA and PSA (Figure 2B,C). PCNA expression is tightly linked to G1/S checkpoint where CDK4-Cyclin D and CDK2-Cyclin E kinase complexes regulate E2F1 transcription factor activation [15]. As shown in Figure 2D, higher protein expression of Cyclin E/A-CDK2 and Cyclin D-CDK4 was significantly suppressed by COFE treatment. In addition, COFE treatments significantly inhibited RB phosphorylation and E2F1 overexpression compared to that in vehicle-treated BPH-1 cells (Figure 2E). Consistent with data obtained from the Western blot analysis, results from RT-qPCR data revealed that the treatment of BPH-1 cells with COFE downregulated the Cyclin E/A-CDK2 and Cyclin D-CDK4 mRNA expression and regulated RB/E2F1 mRNA expression (Figure 2F).

### 2.3. COFE Suppressed Androgen-Dependent Prostate Enlargement in Testosterone-Treated BPH Rats

The measurement of prostate weight is the common approach to assess the development and progression of BPH in vivo. To evaluate the therapeutic effects of COFE on BPH, rats were administered testosterone for 4 weeks, with or without Fina and COFE. As shown in Table 1, the prostate weight in BPH group significantly increased (1.40 ± 0.24 g) compared to that in Sham group (0.57 ± 0.12 g) (*p* < 0.001). In contrast to the BPH group, administration with COFE 50 and 100 significantly suppressed the increase in prostate weight (1.06 ± 0.11 g and 1.02 ± 0.12 g, respectively). Meanwhile, the total body weight indicated no significant difference among all the groups. Abnormal level of DHT, which is derived from testosterone by 5α–reductase, may have a crucial role in BPH progression [16]. To demonstrate the involvement of androgen in the suppression of prostate enlargement by COFE, we assessed the DHT levels and 5α-reductase 2 mRNA expression. In addition, BPH group showed a significant increase in DHT levels compared to that showed by Sham group, whereas the administration of Fina and COFE significantly suppressed the level of DHT. The results of 5α-reductase 2 mRNA expression analysis were consistent with the data obtained from the analysis of the DHT levels. Data revealed that the administration of Fina and COFE significantly alleviates the testosterone-induced overexpression of 5α-reductase 2.

### 2.4. COFE Improved Histological Changes via Regulation of Anti-Apoptosis Proteins in Testosterone-Treated BPH Rats

**Figure 3:**
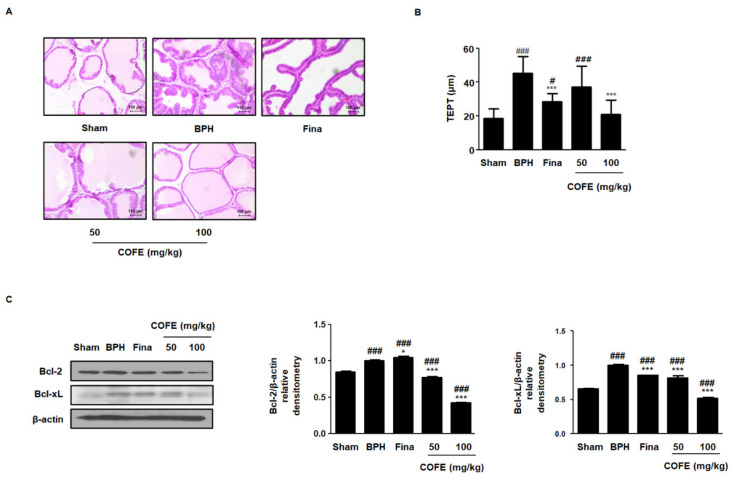
Effects of COFE on cellular proliferation in testosterone-induced BPH rat model. (**A**) Hematoxylin and eosin (H&E) staining was conducted using prostatic tissue sections from testosterone-induced BPH rats model; original magnification 200×. (**B**) Based on H&E staining, thickness of epithelium from prostate tissues (TEPT) was measured and represented as mean ± S.D. of data from three separate prostate tissue sections per group. (**C**) The protein expressions of Bcl2 and Bcl-xL were analyzed using Western blotting using specific antibodies. The protein expression differences were normalized to β-actin. The densitometric analysis of protein bands was performed using Image J Software. Values are mean ± S.D. of data from three separate experiments. ^#^ *p* < 0.05, ^###^ *p* < 0.001 vs. Sham group, * *p* < 0.05, *** *p* < 0.001 vs. BPH group.

Experimental animal model of BPH is characterized by not only increased prostatic weight but also changed histological features. To demonstrate the effects of COFE on morphological changes, we conducted histological analysis using hematoxylin and eosin (H&E) staining and measured the thickness of epithelium from prostate tissues (TEPT). As shown in Figure 3A, the BPH group revealed prostatic hyperplasia signs, such as a thickened epithelium with uninterrupted layer and glandular luminal in comparison to those in the Sham group, whereas the administration of Fina and COFE inhibited the hyperplastic patterns. In addition, COFE 100 administration significantly reduced the level of TEPT following BPH induction (Figure 3B). An imbalance between prostate cell proliferation and apoptosis is linked to the development of BPH [17]. To investigate the effect of COFE on the regulation of apoptosis, we analyzed the expression of anti-apoptotic proteins using Western blotting. In contrast to the Sham group, BPH group showed an overexpression of Bcl-2 and Bcl-xL proteins, whereas COFE exerted dose-dependent inhibitory effects on the protein expression of Bcl-2 and Bcl-xL (Figure 3C).

### 2.5. COFE Suppressed Expression of PCNA in Testosterone-Treated BPH Rats

**Figure 4:**
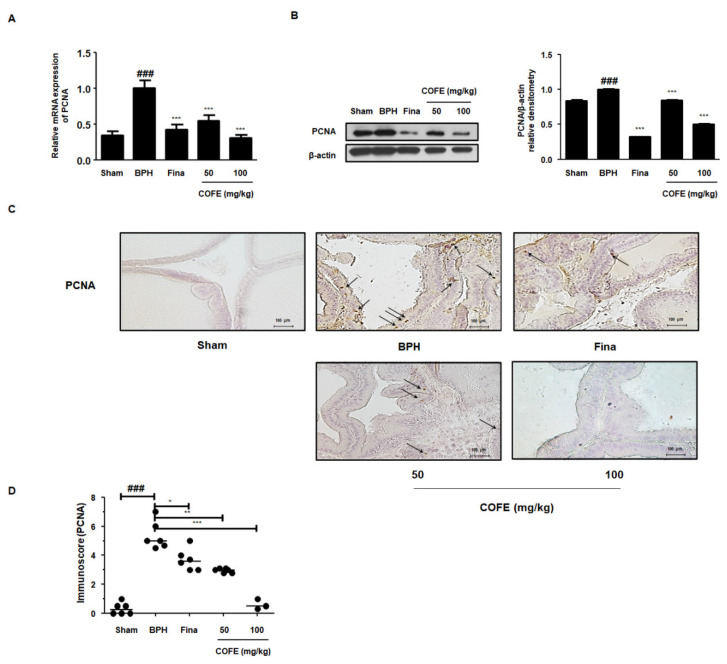
Effects of COFE on PCNA expression in prostate tissues from testosterone-induced BPH rat model. (**A**) The mRNA expression of PCNA in prostate tissues was analyzed by RT-qPCR analysis. The difference of PCNA mRNA expression was normalized to GAPDH. Values are mean ± S.D. of data from three separate experiments. (**B**) The protein expression of PCNA was analyzed by Western blotting using PCNA primary antibody. The protein expression difference was normalized to β-actin. The densitometric analysis was performed using Image J Software. Values are mean ± S.D. of data from three separate experiments. (**C**) The manifestation of PCNA in prostatic tissues from BPH rat was shown by IHC analysis; Original magnification 200×. Arrows, PCNA-positive cells (**D**) Immunoscore of PCNA in prostatic tissue was estimated. Values are mean ± S.D. of data from three separate prostate tissue sections per group. ^###^ *p* < 0.001 vs. Sham group, * *p* < 0.05, ** *p* < 0.01, *** *p* < 0.001 vs. BPH group.

PCNA, a key factor in DNA replication during cell cycle, correlates directly with prostate tissue proliferation [18]. In accordance with in vitro study, RT-qPCR data revealed that COFE administration significantly suppressed the mRNA expression of PCNA compared to that of the testosterone-induced BPH group (Figure 4A). A similar trend was observed in the protein levels. PCNA protein expression in prostatic tissues taken from each experimental group was examined via Western blot and IHC analysis. As shown in Figure 4B,C, the protein expression of PCNA was significantly elevated in BPH group compared to that in Sham group. In contrast, COFE administration clearly downregulated its expression.

### 2.6. COFE Regulated the Cell Cycle Markers in Testosterone-Treated BPH Rats

**Figure 5:**
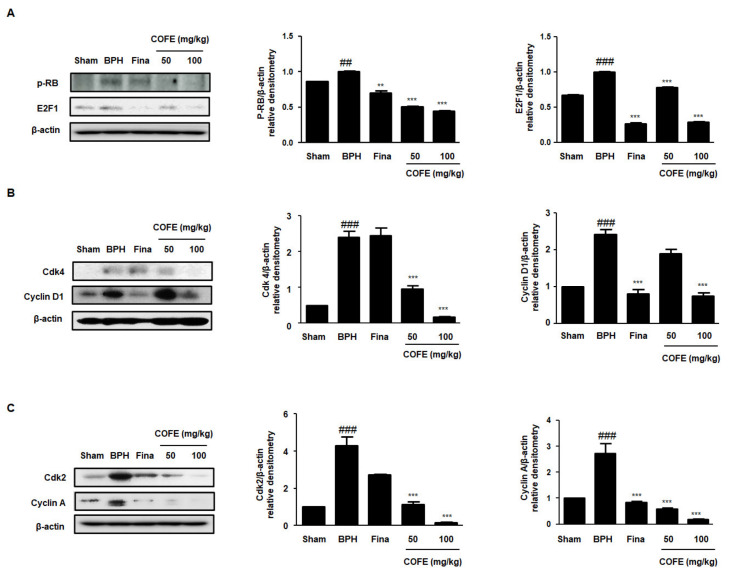
Effects of COFE on p-RB/E2F1 and cell cycle markers in prostate tissues from testosterone-induced BPH rat model. The protein expressions of (**A**) p-RB, E2F1; (**B**) Cdk4, Cyclin D1; and (**C**) Cdk2, and Cyclin A were analyzed by Western blotting using specific antibodies. The protein expression differences were normalized to β-actin. The densitometric analysis was conducted using Image J Software. Values are mean ± S.D. of data from three separate experiments. ^##^ *p* < 0.01, ^###^ *p* < 0.001 vs. Sham group, ** *p* < 0.01, *** *p* < 0.001 vs. BPH group.

From the previous studies, it can be seen that PCNA interacts with Cyclin-CDK complex in several eukaryotic cells [19]. Here, to demonstrate the molecular mechanisms underlying inhibition of cellular proliferation by COFE, we explored the protein expression of cell cycle markers. RB/E2F pathway is generally associated with the regulation of transcription of DNA synthesis and G1/S regulatory genes, such as Cyclin A, Cyclin E, and PCNA [20]. In the BPH group, the phosphorylation of RB and expression of E2F1 protein was significantly increased as compared to those in the Sham group, whereas administration of Fina and COFE significantly suppressed the protein expressions (Figure 5A). As shown in Figure 5B,C, CDK4, Cyclin D1, CDK2, and Cyclin A proteins exhibited an increased expression in the BPH group as compared to that in the Sham group, whereas COFE administration clearly reduced the effect of TP injection. In addition, these results showed that the inhibitory effect of COFE 100 on cell cycle-relative protein expression is superior to that of Fina.

## 3. Discussion

Androgen deprivation therapy is a common primary treatment for BPH. For example, finasteride and dutasteride, both 5α-reductase inhibitors, inhibit the synthesis of DHT by targeting 5α-reductase and are used to halt BPH progression. However, these agents are restricted owing to the adverse effects, such as depression, fatigue, and urinary problems [21,22]. In addition, it has been reported that >17% of patients suffer from BPH progression after long-term treatment of 5α-reductase inhibitors [23]. Androgenic hormones are necessary but not clear contributing factors of BPH [24]. In this regard, recent studies have explored the mechanisms underlying BPH progression, independent of 5α-reductase. We have demonstrated the inhibitory effect on the BPH progression via E2F1/pRB/PCNA-dependent cell cycle signaling pathway regulation [25]. These observations are also supported by other studies that suggest *C. officinalis* as a potential therapeutic agent for BPH treatment [26].

Approximately 300 compounds, including flavonoids, terpenoids, iridoids, tannins, and organic acid, have been isolated and identified from *C. officinalis* [9]. Among them, morroniside and loganin are regarded as standards of content determination for quality control of *C. officinalis*. In the current study, we standardized COFE, determining the persistently high contents of morroniside and loganin (Figure 1). Morroniside and loganin exhibit anti-apoptotic activity on neuron-like cells. In contrast, previous studies have shown that ursolic acid, oleanolic acid, and triterpenoids derived from *C. officinalis*, effectively suppressed the development of BPH in animal model [26,27]. In addition, the aqueous extract of *C. officinalis* inhibited testosterone-induced BPH, raising the intriguing concerns that the pharmacological functions of *C. officinalis* could not be described by only the major components [14]. Although many studies have implied the anti-BPH effect of *C. officinalis* and its compounds, neither the anti-proliferative effect of standardized *C. officinalis* on BPH nor the understanding of molecular mechanism could be considered in much detail.

BPH is a hyperplastic process resulting in the proliferation of epithelial and stromal cells in the transition zone and periurethral area of the prostate [28]. Although the etiology of BPH is complex and poorly understood, several studies have revealed that the androgens play at least a permissive and important role. Both epithelial and stromal cells contain 5α-reductase and androgen receptor involved in steroid metabolism. Most of the DHT is formed in epithelial cells and diffuse to the stromal cells. In stromal cells, DHTs bind to the androgen receptor, stimulating the stromal nuclei producing various growth factors and androgen-dependent genes, which induce the proliferation and differentiation of both epithelial and stromal cells [29]. In this study, we have showed anti-proliferative effects of COFE on BPH and its relative molecular mechanism using epithelial cells (Figure 2). The BPH-1 cell line, derived from human prostate epithelial cells from elderly man with BPH, has been widely used as experimental model for studying prostate growth. As shown in our results, treatment of COFE exerted anti-proliferative effects through the inhibition of pRB–E2F1 signaling pathway by regulating G1 and S checkpoint in prostatic epithelial cells. It has been noted that E2F1 directly interacts with androgen receptor and regulates cellular proliferation mediating the transcription of androgen-responsive gene expressions in prostate cells [30]. To verify the action of COFE on BPH, we established testosterone-induced BPH animal model. Rats with BPH exhibited pathological prostate enlargement, including histological deformity and abnormal level of androgen-relative factors. However, we demonstrated that the administration of COFE significantly alleviated the development of BPH in rats (Table 1). Histologically, the inhibitory effects of COFE on BPH were superior to that of positive control, Fina. The result on the value of TEPT is consistent with our in vitro data, suggesting that anti-BPH effects of COFE may be associated with the inhibitory effect on the prostatic epithelial cells (Figure 3A,B).

Disruption of the balance between proliferation and apoptosis in prostate cells is the mechanism underlying the development of BPH [31]. The Bcl-2 family of proteins is an important component in apoptosis and plays a key role in the development of many type of cancers [32]. It has been noted that the overexpression of Bcl-2 and Bcl-xL, observed in abnormal prostate development, can block Bax activation and mitochondria-mediated cell death [33,34]. In agreement with the mentioned studies, we found Bcl family proteins to be overexpressed in the BPH group, whereas these effects were ameliorated by COFE administration (Figure 3C). Therefore, COFE may exert its inhibitory effects on BPH by recovering the balance between apoptosis and cell survival.

PCNA is an accessory protein in cellular processes, attributing to cell division, DNA replication, and various cell cycle controls [35]. It has been noted that CDK2 could directly interact with PCNA in numerous cells, supporting the linkage between cell cycle checkpoints and PCNA [36]. The G0 to G1 transition is regulated via CDK4–Cyclin D complex and the G2 to S phase transition is regulated via CDK2–cyclin A complex [37]. The results of these studies are consistent with the findings of other studies, in which the PCNA inhibitor induces the arrest of S phase in human prostatic epithelial cancer cell line [38]. As shown in Figure 4, we observed a significant increase in the PCNA gene expression in the prostatic tissues from BPH rats. However, COFE markedly suppressed the overexpression of PCNA in multiple ways. We also demonstrated the inhibitory effects of COFE on the expression of CDK4–Cyclin D and CDK2–Cyclin A proteins in TP-treated BPH rats (Figure 5B,C). These observations support the anti-proliferative effect of COFE on cell cycle progression and on the relationship between PCNA and cell cycle proteins. In addition, consistent with in vitro study, we found that COFE administration showed significant suppressive effects on the E2F1/pRB protein expression in BPH-induced rat model (Figure 5A). Therefore, it can be assumed that COFE administration mediates anti-BPH effect through the inhibition of E2F1 activity and cell cycle progression in prostatic cells.

## 4. Materials and Methods

### 4.1. Chemicals and Reagents

Testosterone propionate (Cat# 200-17532) was purchased from Wako Pure Chemicals (Tokyo, Japan). Finasteride was procured from Merck & Co., Inc. (NJ, USA). Antibodies against Bcl-2 (Cat# sc-7382), Bcl-xL (Cat# sc-8392), CDK2 (Cat# sc-748), CDK4 (Cat# sc-23896), Cyclin A (Cat# sc-751), Cyclin D1 (Cat# sc-753), Cyclin E (Cat# sc-481), E2F1 (Cat# sc-193), PCNA (Cat# sc-56), p-RB (Cat# sc-377528), and β-actin (Cat# sc-81178) were purchased from Santa Cruz biotechnology, Inc. (Dallas, TX, USA). Antibody against PSA (Cat# PB9259) was procured from Boster Biological Technology (Pleasanton, CA, USA). Horseradish peroxidase conjugated secondary antibodies (Cat# 315-035-003 and 111-035-003) were purchased from Jackson ImmunoResearch Laboratories, Inc. (West Grove, PA, USA). SYBR Green Master Mix (Cat# 4367659) was purchased from Applied Biosystems (Foster City, CA, USA). 5α-reductase 2, CDK2, CDK4, Cyclin A, Cyclin D1, Cyclin E, E2F1, PCNA, PSA, RB, and glyceraldehyde-3-phosphate dehydrogenase (GAPDH) oligonucleotide primers were purchased from Bioneer (Daejeon, Korea).

### 4.2. Preparation of the COFE

*C. officinalis* was purchased from Hwapyung D&F Co., Ltd. (Seoul, Korea) and a voucher specimen (No.150510) has been deposited at our laboratory. *C. officinalis* was decocted gently in 10 times volume of 30% (*v*/*v*) ethanol in water for 6 h at 60 °C. The extracts were filtered using 10-μm cartridge paper, and the ethanol was removed using vacuum rotary evaporation (EYELA; Tokyo, Japan). The concentrates were freeze-dried, and the yield was calculated to be 57.91%. The powders were dissolved in distilled water for the experiments, and the residual powder was stored at −20 °C.

### 4.3. Chromatography Conditions

Analyses were performed using a C18 column Luna^®^ (250 × 4.6 I.D. 5 μm particle size, Phenomenex, CA, USA) protected by a disposable security guard precolumn (3.0 × 4.0 mm) and maintained at 35 °C using a thermostatically controlled column heater. The mobile phase was filtered through a polyvinylidene difluoride (PVDF) membrane (0.2 mm, PALL^®^ Corporation, NY, USA) and degassed using an ultrasonic bath. Samples and analytical standard solutions were previously filtered through a 0.2 mm polytetrafluoroethylene membrane (Sartorius^®^, Göttingen, Germany). The mobile phase comprised water containing 10 mM ammonium acetate (pH 6.75, A) and acetonitrile (B). A gradient elution was programmed as follows: 0–5 min, 5.0–5.0% (*v*/*v*) B; 5–55 min, 5–80% B; 55–62 min, 80–90% B; 62–64 min, 90–5% B; and 64–70 min, maintaining 5% B at a flow rate of 1.0 mL/min. Morroniside and loganin were detected at 240 nm. Injection volume was 10 μL.

### 4.4. Calibration Curves, Limits of Detection, and Limits of Quantification

A 70% methanol stock solution containing the two reference components was prepared and diluted to appropriate concentrations for the construction of calibration curves. Two concentrations of the mixed standard solution were injected in triplicates, and their regression values were calculated by the equation Y = AX + B. The results are determined in Table 2. The dilute solution was further diluted to a series of concentrations with 70% methanol for the gain of the limits of detection (LOD) and limits of quantification (LOQ). The LOD and LOQ under the indicated chromatographic conditions were determined at a signal-to-noise (S/N) ratio of 3 and 10, respectively. LOD and LOQ for each analyte are also shown in Supplementary Table S1.

### 4.5. Cell Culture and Sample Treatment

Human BPH epithelial cells, BPH-1 cells, were obtained from the American Type Culture Collection (Manassas, VA, USA). BPH-1 cells were cultured in RPMI 1640 medium (Cat# 11875093, Gibco, MA, USA) containing 20% fetal bovine serum (FBS, Cat# 26140-079) and 100 mg/mL penicillin–streptomycin (Hyclone, Logan, UT, USA). BPH-1 cells were seeded (5 × 10^5^ cells/well) and incubated for 24 h. The cells were treated with various concentrations of COFE (125, 250, 500 μg/mL).

### 4.6. Cell Viability Assays

Cells were treated with the COFE (7.8125–500 μg/mL) and incubated overnight followed by the addition of MTT solution (5 mg/mL) for 4 h. After aspirating the supernatant, the formazan product was dissolved in DMSO, and the extent of cytotoxicity was measured at 570 nm using a BioTek™ Epoch microplate spectrophotometer (Winooski, VT, USA).

### 4.7. Isolation of Total RNA and Reverse Transcription Quantitative Polymerase Chain Reaction (RT-qPCR)

The prostatic cells and prostatic tissues from rats were homogenized, and total RNA was isolated using an Easy-Blue^®^ Reagent (iNtRON Biotechnology, Inc., Gyeonggi-do, Korea). The total RNA samples were quantified with an Epoch^®^ microvolume spectrophotometer system (BioTek Instruments, Inc. Winooski, VT, USA). The cDNA was obtained using isolated total RNA (1 μg), d(T)16 primer, and avian myeloblastosis virus reverse transcriptase (AMV-RT). Relative gene expression was quantified using Real Time PCR System 7500 (Applied Biosystems, Foster City, CA, USA) with SYBR green PCR master mix (Applied Biosystems).

### 4.8. Western Blot Analysis

The prostatic cells and prostatic tissues from rats were homogenized in PRO-PREP^™^ protein extraction solution (Cat# 17061, Intron Biotechnology, Seoul, Korea) and incubated for 15 min at 4 °C. Debris was removed by micro-centrifugation at 11,000× *g*, followed by quick freezing of the supernatants. The protein concentration was determined using the Bio-Rad protein assay reagent according to the manufacturer’s instructions (Bio-Rad, Hercules, CA, USA). Proteins were electro-blotted onto a polyvinylidene difluoride (PVDF) membrane following separation on a 6–12% SDS polyacrylamide gel. The membrane was incubated for 1 h with blocking solution (2.5–5% skim milk) at 24 ± 3 °C, followed by incubation with a 1:1000–2000 diluted primary antibodies overnight at 4 °C. The blots were washed thrice using Tween 20/Tris-buffered saline (T/TBS) and incubated with a secondary antibody for 2 h at 24 ± 3 °C. After washing thrice in T/TBS, the immuno-detection bands were reacted with ECL solution (Cat# RPN2106, Cytiva, Uppsala, Sweden) and recorded on Hyperfilm™ ECL™ (Cat# GE28-9068-37, Cytiva).

### 4.9. Animals

Sprague Dawley rats (8 weeks old, male, 200 ± 20 g) were purchased from DaehanBiolink Co. Ltd. (Daejeon, Korea) and maintained under controlled conditions (temperature, 22 ± 3 °C; humidity, 40–50%; light/dark cycle 12/12 h). Rats were adapted to the feeding conditions for 1 week and then provided free access to food and tap water for 4 weeks. Rats were castrated by intraperitoneal administration of Zoletil^®^ 50 (Virbac, Carros Cedex, France). Rats in the sham-operated group (Sham group; saline 0.2 mL, per oral.) were cut open and sewed up without castration. BPH was induced in rats by subcutaneous injections (s.c.) of testosterone propionate 10 mg/kg/day for 4 weeks after castration. Testosterone-treated rats were divided into four groups (*n* = 8) and orally injected with water (BPH group), finasteride 5 mg/kg (Fina group), COFE 50 and 100 mg/kg (COFE 50 and 100, respectively) for 4 weeks except weekends. On the last day of the 4th week, the animals were fasted for 16 h. The following day, the rats were anesthetized with Zoletil^®^ 50, and the prostatic tissues were excised, weighed, and stored at −80 °C. All procedures were conducted in accordance with the National Institute of Health guidelines and approved by the Institutional Animal Care and Use Committee (IACUC) of the of Sangji University (reg.no. 2015-07).

### 4.10. Serum Concentrations for DHT Analysis

The blood samples were collected and centrifuged at 1000× *g*, for 30 min at 24 ± 3 °C to obtain serum samples, which were immediately frozen at −80 °C for further measurements. Serum concentration of DHT was determined using enzymatic methods with commercial kits (Cusabio Biotech Co Ltd., Houston, TX, USA) according to the manufacturer’s instructions.

### 4.11. H&E Staining

The prostatic samples from rats were fixed in 10% formalin solution and embedded in paraffin. The tissues were cut into 8 mm thickness. The sections were stained with H&E for the histological analysis. Images were acquired using a Leica microscope (Leica DFC295; Wetzlar, Germany).

### 4.12. Immunohistochemistry (IHC)

The 8-mm paraffin sections cut on microscopy slides (Muto pure chemicals co., LTD, Tokyo, Japan) were deparaffinzied and rehydrated using a decreasing ethyl alcohol gradient followed by phosphate buffer saline (PBS). The sections were then blocked with normal goat serum (Fisher scientific, Hampton, VA, USA). The sections were incubated with a 1:200 dilution of primary antibody overnight at 4 °C and washed with PBS. The slides were incubated with a 1:200 dilution of secondary antibody for 30 min at 24 ± 3 °C. After washing thrice in PBS, the slides were incubated with 1 drop (100–200 μL) 3,3′-diaminobenzidine (DAB) solution (abcam^®^, Cambridge, MA, USA) for 1–2 min at 24 ± 3 °C. Finally, the slides were counterstained using hematoxylin solution. Images were acquired using a Leica DM IL LED microscope (Leica^®^, Wetzlar, Germany).

### 4.13. Statistical Analysis

Data are expressed as mean ± standard deviation (SD) of triplicate experiments. Statistical significance was determined using ANOVA and Dunnett’s post hoc test, and *p*-values < 0.05 were considered statistically significant.

## 5. Conclusions

The most significant finding of this study is that COFE has a strong anti-BPH effect via the control of the PCNA/E2F1-dependent cell cycle checkpoint in vivo and in vitro. Moreover, we have identified that the therapeutic effect of COFE on prostatic epithelial cell proliferation is superior to that of finasteride in multiple ways. Therefore, we suggest that COFE could be used as a substitutional agent for BPH treatment.

## Figures and Tables

**Table 1:** Effects of COFE on the androgen-dependent prostatic enlargement in testosterone-induced BPH rat model.

	Index	Sham	BPH	Fina	COFE 50	COFE 100
Group						
Body weight (g)		344.06 ± 26.34	311.17 ± 22.84	300.10 ± 18.88	314.74 ± 24.10	302.14 ± 21.94
Prostate weight (g)		0.58 ± 0.12	1.41 ± 0.25 ^###^	0.97 ± 0.15 ^###,^ ***	1.07 ± 0.11 ^###,^***	1.02 ± 0.12 ^###,^***
Dihydrotestosterone (ng/mL)		1.45 ± 1.22	10.25 ± 1.52 ^###^	6.51 ± 1.22 ^###,^**	6.14 ± 1.01 ^###,^ ***	6.57 ± 0.88 ^###,^**
Relative mRNA expression of 5α-reductase 2		1.06 ± 0.16	3.88 ± 0.80 ^###^	0.76 ± 0.47 ***	1.37 ± 0.64 **	1.15 ± 0.79 **

Values are mean ± S.D. (*n* = 8) of data from three separate experiments. ^###^ *p* < 0.001 vs. Sham group, ** *p* < 0.01, *** *p* < 0.001 vs. BPH group.

**Table 2:** Primer sequences.

Gene Name	Forward Primers (5′-3′)	Reverse Primers (5′-3′)
human PCNA	TTAAACGGTTGCAGGCGTAG	AGGAAAGTCTAGCTGGTTTCGG
human PSA	ATAGGATTGCCCAGGCAGAA	CTAAGGGTAAAAGCAGGGAGAGAGT
human Cyclin E	GACGGGGAGCTCAAAACTGA	TACAACGGAGCCCAGAACAC
human Cyclin A	CGGTACTGAAGTCCGGGAAC	GTTCACAGCCAAATGCAGGG
human CDK2	TTCTATGCCTGATTACAAGCC	CTGGCTTGGTCACATCCT
human Cyclin D1	ACGGCCGAGAAGCTGTGCATC	CCTCCGCCTCTGGCATTTTGGAG
human CDK4	ATGGCTACCTCTCGATATGAGC	CATTGGGGACTCTCACACTCT
human RB	ATGGTTCACCTCGAACACCC	TTTCGACACAACCCTGTCCC
human E2F1	AAGAACCGCTGTTGTCCCG	TCGAGGCCGAAGTGGTAGTC
human β-actin	GGCCAGGTCATCACCATTGG	CTTTGCGGATGTCCACGTCA
rats 5α-reductase 2	GGCAGCTACCAACTGTGACC	CTCCCGACGACACACTCTCT
rats PCNA	CTGCTGGGACATCAGTTCGG	GATCGCAGCGGTATGTGTCG
rats GAPDH	TGATTCTACCCACGGCAAGT	AGCATCACCCCATTTGATGT

## Supplementary Materials

The following are available online at https://www.mdpi.com/1422-0067/21/24/9567/s1.

## Abbreviations

BPH	benign prostatic hyperplasia
COFE	*C. officinalis* 30% ethanol extract
DHT	dihydrotestosterone
PCNA	proliferating cell nuclear antigen
TEPT	thickness of epithelium from prostate tissues
