# Supplementary material for: Anti-Proliferative Effects of Standardized Cornus officinalis on Benign Prostatic Epithelial Cells via the PCNA/E2F1-Dependent Cell Cycle Pathway
Source: Int J Mol Sci. 2020 Dec 15;21(24):9567. doi: 10.3390/ijms21249567 (PMC7765524; doi:10.3390/ijms21249567)
Supplement: Supplementary file 1 [file ijms-21-09567-s001.pdf]

Supplementary Table S1. Regression equation, LODs and LOQs for the two analytes of the assay.

Analyte	Regression Equation	r2	Linear ranage (μg/mL)	LOD(μg/mL)	LOQ(μg/mL)
Morroniside	Y = 16724.4x + 10388.38	0.9999	10.00–100.00	0.2841	0.8610
Loganin	Y = 16559.1x+9546.56	0.9995	20.00–80.00	0.2153	0.6524
